# Secular trends in the epidemiologic patterns of peripheral artery disease and risk factors in China from 1990 to 2019: Findings from the global burden of disease study 2019

**DOI:** 10.3389/fcvm.2022.973592

**Published:** 2022-09-20

**Authors:** Wei Gong, Shuhan Shen, Xiaojing Shi

**Affiliations:** Department of Emergency, The First Affiliated Hospital of Jinzhou Medical University, Jinzhou, China

**Keywords:** peripheral artery disease, epidemiology, China, public health, burden of disease

## Abstract

**Background:**

An understanding of the epidemiologic patterns of peripheral artery disease is essential in public health policy-making. We aimed to assess secular trends in the epidemiologic patterns and risk factors of peripheral artery disease from 1990 to 2019 in China.

**Materials and methods:**

We extracted data on prevalence, incidence, death, and disability-adjusted life years (DALYs) due to peripheral artery disease from the Global Burden of Disease study 2019. In addition, risk factors for peripheral artery disease were reported.

**Results:**

The age-standardized prevalence of peripheral artery disease significantly increased from 1330.42 to 1423.78 per 100,000 population, with an average annual percentage change (AAPC) of 0.16 [95% confidence interval (CI), 0.07 to 0.24] from 1990 to 2019 in China. In addition, the age-standardized mortality rate significantly increased, with an AAPC of 0.62 (95% CI, 0.54 to 0.7), contrasting with the significantly declining trend in age-standardized DALYs (AAPC, −0.45; 95% CI, −0.52 to −0.39) between 1990 and 2019. The age-standardized prevalence was almost three times higher in females than males [2022.13 (95% CI: 1750 to 2309.13) vs. 744.96 (95% CI: 644.62 to 850.82) per 100,000 population] in 2019. The age-specific incidence significantly increased in individuals aged 40–44, 45–49, 50–54, 55–59, and 60–64 years groups but decreased in 70–74, 75–79, and 80–84 years groups. The age and period effects showed that the relative risks of incident peripheral artery disease increased with age and time. The cohort assessment showed that the incidence decreased in successive birth cohorts. Smoking was identified as the risk factor that contributed the most to age-standardized DALYs of peripheral artery disease in 2019.

**Conclusion:**

The burden of peripheral artery disease showed unexpected patterns that varied by age, sex, and year in China. More attention should be given to addressing the increasing incidence among middle-aged individuals and mortality among males.

## Background

Peripheral arterial disease usually refers solely to lower extremity arterial disease, which is characterized by atherosclerosis of the lower extremities leading to arterial stenosis or even occlusion, resulting in chronic or acute ischemic symptoms in lower extremity tissues (1). The onset of the disease is insidious, and there may be no symptoms in the early stage. As the disease progresses, intermittent claudication, ischemic rest pain, ulcers, and long treatment requirements may occur. In severe cases, gangrene may even lead to the need for amputation, and the prognosis is poor. The health hazards of peripheral arterial disease not only stem from the disease itself but also significantly increase the risk of cardiovascular and cerebrovascular events and death in patients, which needs further attention (2, 3). Several studies have shown that peripheral arterial disease is highly prevalent among older adults, and as the global population ages rapidly, the prevalence of peripheral arterial disease is expected to continue to increase (4, 5).

In 2015, there were 236 million people living with peripheral arterial disease worldwide, an increase of 44% compared with 2000, but the awareness, diagnosis, and treatment rates of peripheral arterial disease in this population remained low (6–9). Several previous studies have reported the prevalence of peripheral arterial disease in China, but these studies vary in their survey design, diagnostic criteria, or region (9, 10). Thus, long-term epidemiological information on peripheral arterial disease based on the Chinese population is limited.

Understanding secular trends in epidemiologic patterns of peripheral arterial disease will help guide future research on disease prevention and prevention strategies. The Global Burden of Disease (GBD) study is a global descriptive epidemiological study (11, 12). Therefore, this study aimed to analyze GBD 2019 data to assess the temporal trends in the burden of peripheral arterial disease in China and to explore the net age, period, and cohort effects within an age-period-cohort framework to provide important clues about disease etiology or hypotheses.

## Materials and methods

### Data source

Using all available up-to-date epidemiological data sources and improved standardized methodologies, the GBD 2019 provided a comprehensive assessment of health loss attributed to 369 diseases and injuries and 87 risk factors in 204 countries and territories (11, 12). The sex-stratified age-specific and age-standardized rates and cases of peripheral arterial disease and their 95% confidence intervals (CIs) from 1990 to 2019 in China were directly obtained from the Institute for Health Metrics and Evaluation (IHME) website.^1^ Details of the methodology used in the GBD 2019 have been described in previous studies and are presented in the Supplementary material (11). Data analysis was completed on March 15, 2022. The Institutional Review Board of Jinzhou Medical University determined that this study did not require ethical approval because it used publicly available data.

### Case definition

Individuals with an ankle-brachial index less than 0.9 who were clinically diagnosed with intermittent claudication were defined as having peripheral arterial disease (9). All cardiovascular diseases coded as 440.2, 440.4, and 443.0–443.9 in the 9th revision of the International Classification of Diseases and Injuries (ICD-9) or I70.2–I70.8 and I73–I73.9 in the ICD-10 were recognized as peripheral artery disease (9). More details of the case process are provided in the Supplementary material.

### Statistical analysis

To characterize peripheral artery disease burden by age, sex, and year, descriptive analyses were conducted. The age-specific and age-standardized rates and their average annual percentage changes (AAPCs) were calculated to assess the epidemiologic trends of peripheral artery disease using linear regression analyses, and all rates were reported per 100,000 population (13). The trends of rates were reflected in AAPC values: the rate had an upward trend when the AAPC and the lower boundary of the 95% CI were positive; conversely, the rate had a downward trend when the AAPC and the upper boundary of the 95% CI were negative. In addition, this study applied an age-period-cohort model to assess population risk in a given year and health risk accumulation since birth (14). The methodological details of the age-period-cohort model are described in the Supplementary material. The strength of this model lies in the analysis of the independent effects of age, period, and cohort on the temporal trend of peripheral arterial disease incidence. In this model, the collected data were stratified into successive 5-year age groups and consecutive 5-year periods. Peripheral arterial disease incidence was recorded in successive 5-year age groups (from 40–44 to 85–89 years), consecutive 5-year periods (from 1994 to 2019), and correspondingly consecutive 5-year birth cohorts from 1905–1909 to 1975–1979. The age-period-cohort analysis provided estimated coefficients for age, period, and cohort effects using the intrinsic estimator method. These coefficients were converted to exponential values [exp (coef.) = e*^coef.^*], which represented the relative risk (RR) of incidence for a specific age, period, or birth cohort relative to the sum of the averages for all ages, periods, or birth cohorts. If the RR of the reference group was significantly different from the averages for all ages, periods, or birth cohorts, then two-sided statistical tests and *p* < 0.05 were considered significant. All statistical analyses were performed using GraphPad Prism software (version 8.0) and STATA software (version 15.0).

## Results

**FIGURE 1:**
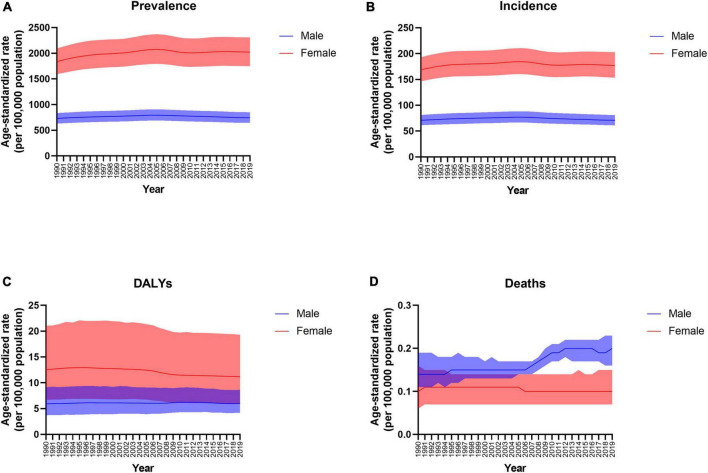
Trends in the age-standardized prevalence **(A)**, incidence **(B)**, disability-adjusted life years (DALYs) **(C)**, and death **(D)** rates of peripheral arterial disease by sex from 1990 to 2019.

As shown in Table 1, the prevalence of the number of cases of peripheral artery disease in China was 28.49 million (95% CI: 24.55 to 32.61 million) in 2019, with an age-standardized prevalence per 100,000 population of 1423.78 (95% CI: 1234.84 to 1625.31), which increased significantly from 1990 (AAPC 0.16, 95% CI: 0.07 to 0.24). Females had a higher age-standardized prevalence and incidence rate than males from 1990 to 2019 (Figures 1A,B). A significant increase in age-specific incidence was observed among individuals aged 40–64 years from 1990 to 2019. The disability-adjusted life years (DALYs) and death rates significantly decreased among females (AAPC in DALYs −0.58, 95% CI: −0.68 to −0.48; AAPC in deaths −0.3, 95% CI: −0.41 to −0.19), but the death rate increased among males (AAPC in deaths 1.45, 95% CI: 1.24 to 1.66) during the last three decades (Figures 1C,D). Although the incidence of peripheral artery disease was higher in 2019 than in 1990 among individuals aged 70–74 years old, the AAPC result indicated a significantly decreasing trend over a period of multiple years with a fluctuating incidence rate.

**TABLE 1:** Sex-and age-specific rates of peripheral artery disease in China in 1990 and in 2019 and their average annual percentage changes (AAPCs) from 1990 to 2019.

Group	Cases (95% CI) in 1990	Rates (95% CI) per 100,000 population in 1990	Cases (95% CI) in 2019	Rates (95% CI) per 100,000 population in 2019	AAPCs in rates (95% CIs), 1990–2019
**Prevalence**					
Age-standardized estimate in overall population	10399944 (8919419–11886890)	1330.42 (1153.94–1516.87)	28489637 (24548557–32612500)	1423.78 (1234.84–1625.31)	0.16 (0.07 to 0.24)*
Age-standardized estimate by sex					
Male	2821530 (2402500–3252853)	731.02 (631.44–836.51)	7174204 (6129082–8248961)	744.96 (644.62–850.82)	0.03 (−0.07 to 0.13)
Female	7578414 (6504359–8678021)	1839.43 (1593.06–2095.46)	21315433 (18333833–24451721)	2022.13 (1750–2309.13)	0.23 (0.14 to 0.32)*
**Age-specific estimate by age group**					
40–44	320920 (237926–420858)	477.26 (353.83–625.88)	515075 (383186–668588)	506.74 (376.98–657.76)	0.16 (0.03 to 0.28)*
45–49	577311 (447627–727652)	1116.23 (865.48–1406.91)	1451259 (1124451–1830389)	1195.77 (926.49–1508.15)	0.19 (0.06 to 0.32)*
50–54	989865 (749247–1279048)	2070.76 (1567.4–2675.72)	2815441 (2144460–3630332)	2250.49 (1714.15–2901.87)	0.24 (0.11 to 0.36)*
55–59	1395461 (1093067–1699100)	3210.72 (2514.96–3909.34)	3323665 (2607704–4037459)	3504.51 (2749.6–4257.15)	0.25 (0.15 to 0.36)*
60–64	1583647 (1244772–1929183)	4470.98 (3514.26–5446.5)	3841863 (3001930–4669422)	4890.61 (3821.4–5944.08)	0.27 (0.19 to 0.34)*
65–69	1696022 (1380960–2057448)	6197.22 (5045.99–7517.86)	4733193 (3852150–5763069)	6724.84 (5473.07–8188.07)	0.19 (0.13 to 0.26)*
70–74	1565287 (1236166–1932352)	8301.08 (6555.68–10247.72)	4209488 (3321698–5204012)	8796.17 (6941.04–10874.33)	0.09 (0.02 to 0.16)*
75–79	1196868 (970541–1454484)	10489.2 (8505.7–12746.91)	3255649 (2641880–3941281)	10907.97 (8851.55–13205.17)	0.01 (−0.07 to 0.08)
80–84	715784 (588286–865214)	12693.72 (10432.67–15343.71)	2499705 (2053349–3021157)	13109.85 (10768.91–15844.64)	0 (−0.08 to 0.09)
85+	358781 (298780–423108)	15251.29 (12700.73–17985.75)	1844299 (1535687–2178011)	16474.22 (13717.54–19455.11)	0.22 (0.16 to 0.28)*
**Incidence**					
Age-standardized estimate in overall population	1029594 (887381–1177955)	121.45 (105.55–138.63)	2615880 (2251154–3008605)	125.43 (109.07–143.36)	0.02 (−0.05 to 0.1)
**Age-standardized estimate by sex**					
Male	295238 (252935–342299)	70.98 (61.43–81.15)	711273 (609570–819828)	71.01 (61.21–81.32)	−0.05 (−0.17 to 0.06)
Female	734356 (632742–842692)	168.9 (146.61–192.84)	1904608 (1643535–2191217)	177.25 (153.51–202.97)	0.06 (−0.02 to 0.14)
**Age-specific estimate by age group**					
40–44	63114 (48739–80050)	93.86 (72.48–119.05)	81655 (59423–110422)	99.53 (77.02–126.01)	0.15 (0.02 to 0.28)*
45–49	87755 (57694–123753)	169.67 (111.55–239.28)	101164 (78288–128088)	180.93 (119.3–254.47)	0.17 (0.04 to 0.31)*
50–54	119123 (88583–150694)	249.2 (185.31–315.25)	219588 (144795–308835)	266.35 (198.21–335.92)	0.17 (0.06 to 0.28)*
55–59	144831 (91023–200859)	333.23 (209.43–462.14)	333213 (247966–420253)	351.76 (218.07–490.85)	0.11 (0.04 to 0.19)*
60–64	160906 (119095–206521)	454.27 (336.23–583.05)	333604 (206821–465520)	475.28 (349.06–612.64)	0.08 (0.01 to 0.15)*
65–69	168516 (111895–240140)	615.75 (408.86–877.46)	373362 (274205–481268)	638.53 (418.34–910.83)	0.01 (−0.07 to 0.08)
70–74	135947 (97596–182745)	720.96 (517.57–969.14)	449419 (294440–641079)	729.54 (522.11–974.55)	−0.08 (−0.16 to −0.01)*
75–79	88389 (60446–121235)	774.63 (529.74–1062.49)	349127 (249859–466380)	768.17 (522.01–1046.56)	−0.16 (−0.23 to −0.08)*
80–84	43782 (30480–60805)	776.43 (540.53–1078.31)	229273 (155801–312361)	762.96 (525.48–1057.74)	−0.16 (−0.23 to −0.08)*
85+	17231 (12460–23092)	732.45 (529.64–981.6)	145476 (100195–201684)	729.38 (530.79–986.35)	−0.04 (−0.09 to 0.01)
**DALYs**					
Age-standardized estimate in overall population	67569 (37810–110283)	9.65 (5.41–15.99)	165729 (96082–274013)	8.82 (5.14–14.4)	−0.45 (−0.52 to −0.39)*
**Age-standardized estimate by sex**					
Male	19530 (12081–30169)	5.92 (3.72–9.15)	51953 (35758–76979)	6 (4.2–8.63)	0.03 (−0.03 to 0.09)
Female	48039 (25167–80776)	12.48 (6.68–21.06)	113776 (59746–197732)	11.17 (5.92–19.28)	−0.58 (−0.68 to −0.48)*
**Age-specific estimate by age group**					
40–44	724 (519–1043)	1.08 (0.77–1.55)	967 (773–1201)	0.95 (0.76–1.18)	−0.82 (−0.97 to −0.67)*
45–49	655 (484–963)	1.27 (0.94–1.86)	1508 (1170–1925)	1.24 (0.96–1.59)	−0.1 (−0.26 to 0.07)
50–54	1814 (1166–2785)	3.79 (2.44–5.83)	4281 (2938–6377)	3.42 (2.35–5.1)	−0.48 (−0.56 to −0.41)*
55–59	4935 (2612–8530)	11.35 (6.01–19.63)	9461 (5078–16084)	9.98 (5.35–16.96)	−0.61 (−0.7 to −0.51)*
60–64	8708 (4503–15070)	24.59 (12.71–42.55)	17164 (9114–29542)	21.85 (11.6–37.61)	−0.55 (−0.62 to −0.47)*
65–69	13188 (6879–24248)	48.19 (25.14–88.6)	30393 (16281–55505)	43.18 (23.13–78.86)	−0.52 (−0.59 to −0.44)*
70–74	14494 (7464–25525)	76.86 (39.58–135.37)	32970 (17890–56996)	68.89 (37.38–119.1)	−0.53 (−0.61 to −0.46)*
75–79	11796 (6074–20910)	103.38 (53.23–183.25)	27903 (15066–50942)	93.49 (50.48–170.68)	−0.51 (−0.57 to −0.44)*
80–84	7420 (3941–12708)	131.59 (69.88–225.36)	23134 (13273–40169)	121.33 (69.61–210.67)	−0.42 (−0.47 to −0.36)*
85+	3835 (2191–6454)	163.04 (93.14–274.34)	17949 (10586–29332)	160.33 (94.56–262)	−0.17 (−0.23 to −0.11)*
**Deaths**					
Age-standardized estimate in overall population	609 (480–826)	0.12 (0.09–0.16)	2209 (1810–2745)	0.14 (0.11–0.18)	0.62 (0.54 to 0.7)*
**Age-standardized estimate by sex**					
Male	300 (230–395)	0.14 (0.11–0.19)	1293 (1040–1551)	0.2 (0.16–0.23)	1.45 (1.24 to 1.66)*
Female	310 (179–480)	0.1 (0.06–0.16)	917 (617–1363)	0.1 (0.07–0.15)	−0.3 (−0.41 to −0.19)*
**Age-specific estimate by age group**					
40–44	15 (11–22)	0.02 (0.02–0.03)	21 (16–26)	0.02 (0.02–0.03)	−0.82 (−0.97 to −0.67)*
45–49	15 (11–23)	0.03 (0.02–0.04)	35 (27–44)	0.03 (0.02–0.04)	−0.08 (−0.25 to 0.09)
50–54	24 (17–34)	0.05 (0.04–0.07)	59 (46–75)	0.05 (0.04–0.06)	−0.21 (−0.29 to −0.13)*
55–59	36 (27–51)	0.08 (0.06–0.12)	75 (59–96)	0.08 (0.06–0.1)	−0.24 (−0.32 to −0.15)*
60–64	48 (36–68)	0.13 (0.1–0.19)	109 (87–136)	0.14 (0.11–0.17)	0.07 (−0.03 to 0.17)
65–69	69 (54–97)	0.25 (0.2–0.35)	202 (161–250)	0.29 (0.23–0.35)	0.51 (0.4 to 0.62)*
70–74	95 (74–133)	0.5 (0.39–0.7)	290 (234–351)	0.61 (0.49–0.73)	0.68 (0.58 to 0.79)*
75–79	104 (82–144)	0.92 (0.72–1.27)	340 (278–417)	1.14 (0.93–1.4)	0.93 (0.81 to 1.04)*
80–84	104 (80–138)	1.85 (1.41–2.45)	454 (369–563)	2.38 (1.94–2.95)	1.06 (0.86 to 1.26)*
85+	98 (68–132)	4.16 (2.9–5.6)	624 (484–837)	5.58 (4.32–7.48)	0.91 (0.77 to 1.04)*

*Indicates a *p* value < 0.05.

The estimated RRs of peripheral artery disease incidence according to age, period, and cohort effects are shown in Table 2. When the period and cohort effects were controlled for, the RR of peripheral artery disease incidence significantly increased with advancing age. Compared with the average RRs of all age groups, a significantly increased RR of peripheral artery disease incidence was observed in the 60-year-old and older groups among both males and females. Regarding the period effect, we observed upward trends in the risk of developing peripheral artery disease among both males and females. The RRs associated with period effects showed significant increases from 0.87 (95% CI, 0.84–0.9) in 1994 to 1.1 (95% CI, 1.06–1.14) in 2019 for males and a significantly higher RR of 1.11 (95% CI, 1.09–1.14) in 2019 for females compared with the average RRs of all periods (Table 2). Birth cohort effects had a significant effect on peripheral artery disease incidence (Table 2). Compared with the average RRs of all cohorts, the RRs continuously decreased with later birth cohorts for both males and females, especially after the 1950–1954 birth cohort.

**TABLE 2:** Sex-specific relative risks of peripheral artery disease incidence in China due to age, period, and cohort effects.

Factor	Males		Females	
	RR (95% CI)	*P*-value	RR (95% CI)	*P*-value
**Age**				
40–44	0.3 (0.28–0.34)	< 0.001	0.31 (0.29–0.33)	< 0.001
45–49	0.49 (0.45–0.52)	< 0.001	0.55 (0.53–0.58)	< 0.001
50–54	0.69 (0.65–0.73)	< 0.001	0.75 (0.72–0.78)	< 0.001
55–59	0.9 (0.85–0.95)	< 0.001	0.92 (0.89–0.96)	< 0.001
60–64	1.15 (1.1–1.21)	< 0.001	1.2 (1.16–1.24)	< 0.001
65–69	1.44 (1.37–1.5)	< 0.001	1.54 (1.49–1.58)	< 0.001
70–74	1.58 (1.52–1.65)	< 0.001	1.66 (1.62–1.7)	< 0.001
75–79	1.62 (1.56–1.69)	< 0.001	1.62 (1.58–1.66)	< 0.001
80–84	1.62 (1.56–1.68)	< 0.001	1.45 (1.41–1.48)	< 0.001
85–89	1.58 (1.52–1.65)	< 0.001	1.19 (1.15–1.22)	< 0.001
**Period**				
1994	0.87 (0.84–0.9)	< 0.001	0.88 (0.86–0.9)	< 0.001
1999	0.94 (0.91–0.98)	0.001	0.94 (0.92–0.96)	< 0.001
2004	1.02 (0.98–1.05)	0.371	1 (0.98–1.02)	0.875
2009	1.03 (1–1.07)	0.057	1.02 (1–1.04)	0.122
2014	1.06 (1.03–1.1)	< 0.001	1.07 (1.05–1.09)	< 0.001
2019	1.1 (1.06–1.14)	< 0.001	1.11 (1.09–1.14)	< 0.001
**Cohort**				
1905–1909	1.43 (1.31–1.57)	< 0.001	1.41 (1.32–1.5)	< 0.001
1910–1914	1.37 (1.28–1.46)	< 0.001	1.34 (1.28–1.4)	< 0.001
1915–1919	1.3 (1.23–1.37)	< 0.001	1.27 (1.22–1.32)	< 0.001
1920–1924	1.24 (1.18–1.3)	< 0.001	1.21 (1.17–1.25)	< 0.001
1925–1929	1.18 (1.13–1.24)	< 0.001	1.15 (1.11–1.19)	< 0.001
1930–1934	1.12 (1.07–1.17)	< 0.001	1.09 (1.06–1.13)	< 0.001
1935–1939	1.06 (1.01–1.11)	0.028	1.04 (1.01–1.08)	0.012
1940–1944	1 (0.94–1.06)	0.945	0.99 (0.96–1.03)	0.74
1945–1949	0.94 (0.89–1)	0.059	0.95 (0.91–0.99)	0.006
1950–1954	0.9 (0.84–0.96)	0.001	0.91 (0.87–0.95)	< 0.001
1955–1959	0.85 (0.79–0.92)	< 0.001	0.87 (0.83–0.92)	< 0.001
1960–1964	0.81 (0.75–0.89)	< 0.001	0.84 (0.79–0.88)	< 0.001
1965–1969	0.77 (0.7–0.86)	< 0.001	0.79 (0.74–0.84)	< 0.001
1970–1974	0.73 (0.63–0.83)	< 0.001	0.75 (0.69–0.81)	< 0.001
1975–1979	0.69 (0.53–0.89)	0.005	0.71 (0.6–0.84)	< 0.001

RR denotes the relative risk of peripheral artery disease incidence in a particular age, period, or birth cohort relative to the average RRs of all age, period, or birth cohort combined. RR, relative risk; CI, confidence interval.

**FIGURE 2:**
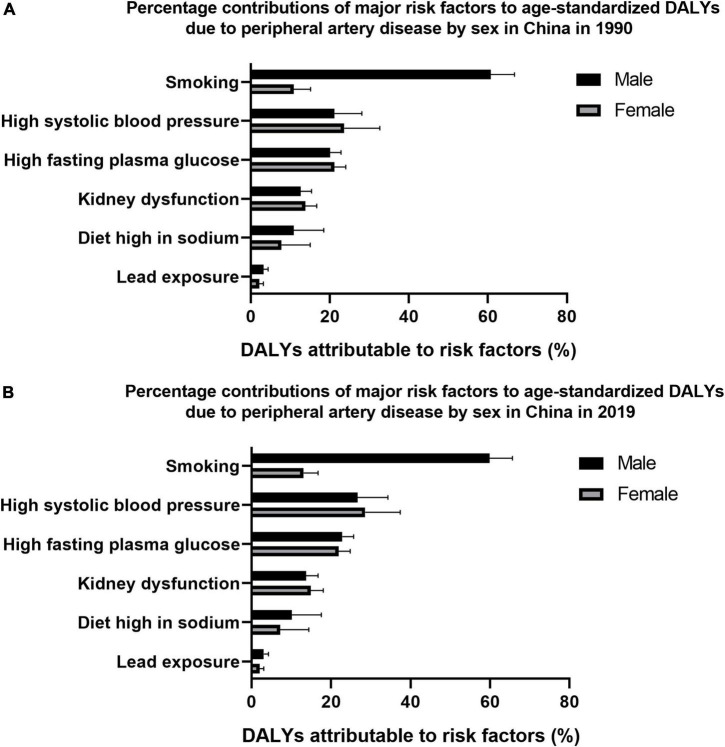
Percentage contributions of major risk factors to age-standardized disability-adjusted life years (DALYs) due to peripheral artery disease by sex in China in 1990 **(A)** and 2019 **(B)**.

Figure 2 reflects all associated risk factors quantified in the GBD 2019. The age-standardized DALYs of peripheral artery disease were primarily attributable to smoking [28.1% (95% CI 22.6% to 32.9%)], followed by high systolic blood pressure [27.9% (95% CI 20.4% to 35.3%)], high fasting plasma glucose [22.1% (95% CI 19.4% to 24.9%)], kidney dysfunction [14.3% (95% CI 11.2% to 17.5%)], high-sodium diet [8.1% (95% CI 2.8% to 15.2%)], and lead exposure [2.4% (95% CI 1.4% to 3.5%)] in China in 2019. The top attributable risk factor for DALYs of peripheral artery disease for males was smoking [59.9% (95% CI 51.3% to 65.6%)] and for females was high systolic blood pressure [28.6% (95% CI 20.3% to 37.4%)] in 2019. The percentage contributions of smoking was 60.8% (95% CI 51.8% to 66.8%) for males and the percentage contributions of high systolic blood pressure was 23.7% (95% CI, 15.4% to 32.6%) for females in 1990.

## Discussion

To our knowledge, the present study is the first comprehensive assessment of the epidemiologic patterns of peripheral arterial disease in China over the past three decades using GBD 2019 data. This study found that between 1990 and 2019, the standardized prevalence of peripheral arterial disease increased significantly among females, and the incidence increased significantly among individuals aged 40–64 years old. In addition, standardized DALYs and mortality due to peripheral arterial disease were significantly decreased among females, but the standardized mortality significantly increased among males.

This study expands on previous research by including the incidence, mortality, and DALYs of peripheral arterial disease instead of focusing only on prevalence. The patterns and trends were heterogeneous across sexes, which illustrates the necessity of resource allocation by sex and priority setting in China. Since the data from the GBD 2019 featured by its unified and standardized approach involve the latest advancements in data analytical techniques, these estimates are highly comparable over the 1990 to 2019 range (11, 12). In addition, this study provides an example of systematic mining of disease trends using GBD data. For other non-communicable chronic diseases, the application of age-period-cohort models to estimate disease trends by age, period, and cohort may also provide a clearer picture of the effectiveness of health system responses beyond traditional epidemiological indicators, thus contributing to the early achievement of the Healthy China 2030 goals.

A meta-analysis study reported that the number of people with peripheral arterial disease would increase by 40% in China between 2000 and 2020, with approximately 41 million Chinese adults projected to be affected by peripheral arterial disease in 2020 (10). Although the current prevalence trend is consistent with the results of that previous study, the predicted number of patients was higher than that in the GBD, which may be because the previous result was highly dependent on the assumptions of the statistical model, but in fact, the epidemiological pattern of peripheral arterial disease is constantly changing. This study found a higher prevalence of disease among females than males, which is consistent with several previous studies (6, 10). However, a cross-sectional study in China did not find such sex differences, which may be explained by the fact that the previous study adjusted for confounding factors, including sociodemographic factors, metabolic disease, and cardiovascular disease, to compare differences between males and females, resulting in no significant differences (9). Although the reasons for the higher prevalence and incidence of peripheral arterial disease in females are unknown, the mechanisms may be multifactorial. It is estimated that more than half of female non-smokers in China are exposed to passive smoking, which may be one of the possible mechanisms (15). This paradoxical female preponderance of peripheral artery disease might be inherent to the disease mechanism or a combined effect of other potential risk factors in developing countries, such as obesity and socioeconomic inequality (16–18).

Previous studies have shown that peripheral arterial disease is widespread among elderly individuals (6). Epidemiological surveys in China indicate that the prevalence of peripheral arterial disease in the general population over 35 years of age is 6%, and the prevalence of peripheral arterial disease among elderly individuals over 60 years of age is 15% (9, 10). It is estimated that there are more than 30 million patients living with peripheral arterial disease in China. Moreover, with the accelerated aging of the population, it can be expected that aging will lead to a rapid increase in the prevalence of peripheral arterial disease based on China’s very large population base, causing very large social and economic burdens and becoming a major public health and social issue in China that urgently needs to be solved.

Notably, the mortality of peripheral arterial disease continued to increase among males. The increase in overall mortality was mainly contributed by the increased mortality burden on males. Many factors, such as population aging and a higher increase in rates of non-communicable chronic diseases among males, may have contributed to this rise in mortality (19, 20). A previous study has indicated that males had more years of life lost associated with population aging than females in the last three decades (20). We observed a decline in mortality due to peripheral arterial disease among females over the past three decades, further prolonging survival time, which may have contributed to the continued rise in the prevalence of peripheral arterial disease among females. Previous studies have reported significant sex differences in peripheral arterial disease (6, 21). This sex difference in peripheral arterial disease manifestations may stem from a sex dimorphism in healthy vascular stroma and sex differences in responses to vascular stressors (21). The current study found that DALYs in the overall population showed a downward trend, and the decline in this index was contributed by females. Although the reasons for the reduced years lived with peripheral arterial disease disability are not clear, the decreased years of life lost among females could be explained by a reduction in mortality. However, these sex differences may be related to unknown risk factors or represent a survival advantage for females, with males being more likely to die from concomitant coronary heart disease (7). A previous study confirmed that current smoking, hypertension, and obesity are highly associated with peripheral arterial disease mortality (19). It is estimated that the prevalence of smoking among males is 17 times higher than that among females in China, and the prevalence of hypertension among males increased significantly from 2007 to 2017, but no significant change was observed among females, which may further explain the higher mortality rate of peripheral arterial disease among males than among females (22, 23). In addition, the prevalence of smoking among females in China decreased from 3.1% in 2003 to 2.7% in 2013, which may reduce peripheral arterial disease mortality among females (22). Furthermore, the prevalence of overweight among Chinese females decreased significantly from 2007 to 2017, which may also contribute to the reduction in peripheral arterial disease mortality among females (23). Although there are proven treatments to decrease the risk of death in patients diagnosed with peripheral arterial disease, significant sex differences in treatment and outcomes have been observed (21). Future studies aimed at reducing the mortality burden among males are needed.

The age-period-cohort model has become a popular method to assess the impact of chronological age, time period, and birth cohort on morbidity (14). The age effect represented a different risk of outcomes across age groups. This study revealed that the risk began to increase significantly after the age of 60 years for both males and females. These results are supported by previous studies (10). The current study suggests that the increase in the burden of peripheral artery disease is due to an increase in the number of surviving peripheral artery disease patients, the primary reason for which may be the contribution of population aging (24). Due to the rapid aging of China’s population, the burden of peripheral artery disease may continue to increase in the future. To prevent social costs from surging in the future, this study suggests that people over the age of 60 years should be actively screened for this disease during routine physical examinations.

Period effects reflect the influence of a series of complex historical events and environmental factors (14). The incidence of peripheral arterial disease has increased substantially since 2014, which may be related to the rapid development of China’s economy and changes in the lifestyle and behavioral habits of the Chinese population, including unhealthy dietary and sedentary habits (25). These changes increase the risk of developing chronic diseases such as diabetes and hypertension, which are significantly associated with the development of peripheral arterial disease (6). In addition, studies have reported a significant increase in the prevalence of diabetes and hypertension in China in the past decade (23, 24, 26). Birth cohort effects reflect the characteristics of each generation and take into account exposure to risk factors and environmental factors early in life (14). The possible reason for the cohort-related findings in this study is that the cohort born later had better education and a higher awareness of health and disease prevention than the early-born cohort (27). Although peripheral arterial disease poses a very large and increasing burden and threat to the health care system and society, it remains an underappreciated problem (28, 29). In addition to the need for more comprehensive planning and implementation of clinical services, greater emphasis should be placed on prevention strategies, such as early education and public outreach for peripheral arterial disease.

Previous studies have reported that smoking, hypertension, diabetes, and dyslipidemia can increase the risk of peripheral arterial disease and are independent risk factors for peripheral arterial disease (30, 31). The current study found that the risk factor that contributed the most to DALYs was smoking. Studies have shown that smoking still significantly increases the prevalence of peripheral arterial disease among people who quit smoking within the past 10 years, while smoking cessation for more than 10 years has no effect on peripheral arterial disease (30, 32). Notably, the risk factor that contributed the most among women was high blood pressure. Hypertension is one of the main factors leading to the development of peripheral arterial disease, which frequently presents as asymptomatic ischemia before the onset of ischemic symptoms such as intermittent claudication or lower extremity pain. Therefore, it is necessary to strengthen screening and interventions for such populations.

These findings provide epidemiological evidence for the increased burden of peripheral arterial disease in China. However, this study has some limitations. First, the age-period-cohort model treats a community as a unit of observation and analysis, which can lead to ecological fallacy. Second, although ICD codes are widely accepted for use on death certificates and the GBD 2019 made significant efforts to improve data quality, misclassification of peripheral arterial disease deaths cannot be completely avoided because peripheral arterial disease is often accompanied by comorbidities such as myocardial infarction or stroke (11). Third, an estimate of peripheral arterial disease burden is obtained using a combination of data, which depends heavily on the quality and quantity of data used in the modeling process. Fourth, although unified diagnostic criteria were used in this study, there may be differences in measurement methods between studies, which may affect the accuracy of the results. Therefore, our findings on the epidemiology of peripheral arterial disease in this study should be interpreted with caution.

## Conclusion

In conclusion, peripheral arterial disease continues to be an increasingly serious public health issue in China, with considerable heterogeneity in its epidemiological pattern across age and sex groups. Despite great strides in peripheral arterial disease prevention and treatment, the incidence of peripheral arterial disease among middle-aged adults, as well as mortality among men, increased significantly from 1990 to 2019. More strategies should be used to implement cost-effective interventions and address modifiable risk factors, especially among middle-aged individuals and males.

## Data availability statement

The original contributions presented in this study are included in the article/Supplementary material, further inquiries can be directed to the corresponding author.

## Ethics statement

Ethical review and approval was not required for this study in accordance with the local legislation and institutional requirements. Written informed consent was not required for this study in accordance with the local legislation and institutional requirements.

## Author contributions

WG contributed to the data acquisition, analysis, interpretation and drafted, and critically reviewed the manuscript for intellectual content. WG and SS contributed to the data analysis. WG and XS conceived and designed the study. XS was the guarantor of this work and, as such, had full access to all the data in the study and takes responsibility for the integrity of the data and the accuracy of the data analysis. All authors reviewed and approved the final version of the manuscript.

## Footnotes

1
https://vizhub.healthdata.org/gbd-results
