# Supplementary material for: Secular trends in the epidemiologic patterns of peripheral artery disease and risk factors in China from 1990 to 2019: Findings from the global burden of disease study 2019
Source: Front Cardiovasc Med. 2022 Sep 20;9:973592. doi: 10.3389/fcvm.2022.973592 (PMC9530250; doi:10.3389/fcvm.2022.973592)
Supplement: Supplementary file 1 [file Data_Sheet_1.docx]

**Supplementary Materials**

Supplement 1. Overview, data sources, modeling, definition, and data process for Global Burden of Disease 2019.

Supplement 2. Methodological details of the age-period-cohort model.

**Supplement 1. Overview, data source, and modeling for Global Burden of Disease 2019**

**Overview**

The Global Burden of Disease (GBD) is an approach to global descriptive epidemiology.^1^ It is a systematic, scientific effort to quantify the comparative magnitude of health loss due to diseases, injuries, and risk factors by age, sex, and geography for specific points in time. Institute for Health Metrics and Evaluation (IHME) serves as the coordinating center for the GBD and affiliated projects. Published in The Lancet in October 2020, GBD 2019 provides, for the first time, an independent estimation of population for each of 204 countries and territories and for the globe using a standardized, replicable approach, as well as a comprehensive update on fertility and migration.^1^ GBD 2019 incorporates major data additions and improvements and methodological refinements. Mortality and life expectancy estimates have expanded to a total of 990 locations at the most detailed level, and new causes have been added to the fatal and nonfatal cause lists, for a total of 369 diseases and injuries (http://www.healthdata.org/gbd/about/protocol). GBD 2019 estimated each epidemiological quantity of interest—incidence, prevalence, mortality, years lived with disability (YLDs), years of life lost (YLLs), and disability-adjusted life-years (DALYs)—for 23 age groups; males, females, and both sexes combined; and 204 countries and territories that were grouped into 21 regions and seven super-regions. The GBD 2019 location hierarchy now includes all WHO member states. The GBD disease and injury analytical framework generated estimates for every year from 1990 to 2019. Diseases and injuries were organized into a levelled cause hierarchy from the three broadest causes of death and disability at Level 1 to the most specific causes at Level 4. Within the three Level 1 causes—communicable, maternal, neonatal, and nutritional diseases; noncommunicable diseases; and injuries—there are 22 Level 2 causes, 174 Level 3 causes, and 301 Level 4 causes (including 131 Level 3 causes that are not further disaggregated at Level 4). In total, 364 causes are nonfatal and 286 are fatal.^1^

**Data sources**

The GBD estimation process is based on identifying multiple relevant data sources for each disease or injury, including censuses, household surveys, civil registration and vital statistics, disease registries, health service use, air pollution monitors, satellite imaging, disease notifications, and other sources. Each of these types of data is identified from a systematic review of published studies, searches of government and international organization websites, published reports, primary data sources such as the Demographic and Health Surveys, and contributions of datasets by GBD collaborators. Aa total of 86,249 sources were used in this analysis, including 19,354 sources reporting deaths, 31,499 reporting incidence, 1973 reporting prevalence, and 26,631 reporting other metrics. Each newly identified and obtained data source is given a unique identifier by a team of librarians and included in the Global Health Data Exchange (GHDx). The GHDx makes publicly available the metadata for each source included in GBD as well as the data, where allowed by the data provider. Additional metadata for each source are available in the online GBD citation tool, http://ghdx.healthdata.org/gbd-results-tool.

**Modeling**

For most diseases and injuries, processed data are modeled using standardized tools to generate estimates of each quantity of interest by age, sex, location, and year.^1^ There are three main standardized tools: the cause of death ensemble model (CODEm), spatiotemporal Gaussian process regression (ST-GPR), and DisMod-MR. Previous publications provide more details on these general GBD methods.^2-4^ Briefly, CODEm is a highly systematized tool to analyze cause of death data using an ensemble of different modeling methods for rates or cause fractions with varying choices of covariates that perform best with out-of-sample predictive validity testing. DisMod-MR is a Bayesian meta-regression tool that allows evaluation of all available data on incidence, prevalence, remission, and mortality for a disease, enforcing consistency between epidemiological parameters. Previous studies showed that DisMod­MR can produce robust and valid estimates compared with real surveillance data.^5^ ST-GPR is a set of regression methods that borrow strength between locations and over time for single metrics of interest, such as risk factor exposure or mortality rates.^1^

**Definition and data process**

Individuals with an ankle-brachial index less than 0.9 who were clinically diagnosed with intermittent claudication were defined as having peripheral arterial disease.


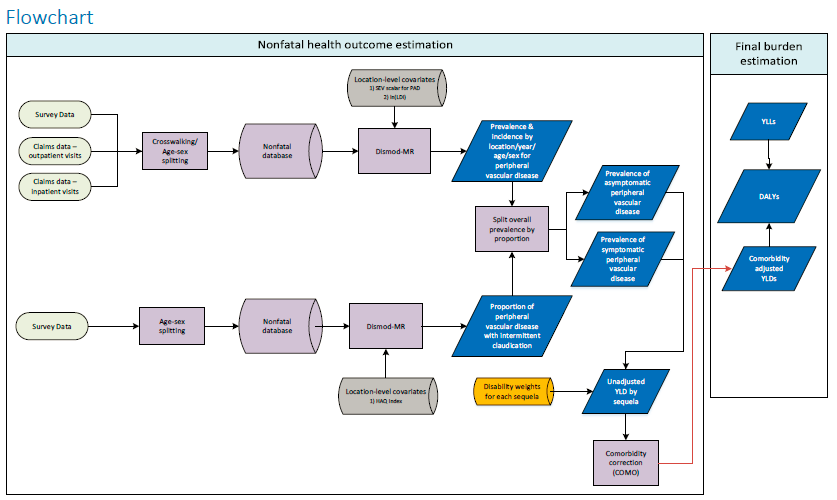


Estimation process for DALYs

To estimate DALYs for GBD 2019, we started by estimating cause-specific mortality and non‐fatal health loss. For each year for which YLDs have been estimated, we computed DALYs by adding YLLs and YLDs for each age-sex-location. Uncertainty in YLLs was assumed to be independent of uncertainty in YLDs. We calculated 1000 draws for DALYs by summing the first draw of the 1000 draws for YLLs and YLDs and then repeating for each subsequent draw. 95% UIs were computed by using the 25th and 975th ordered draw of the DALY uncertainty distribution. We calculated DALYs as the sum of YLLs and YLDs for each cause, location, age group, sex, and year. For more information, please refer to the following figure A.


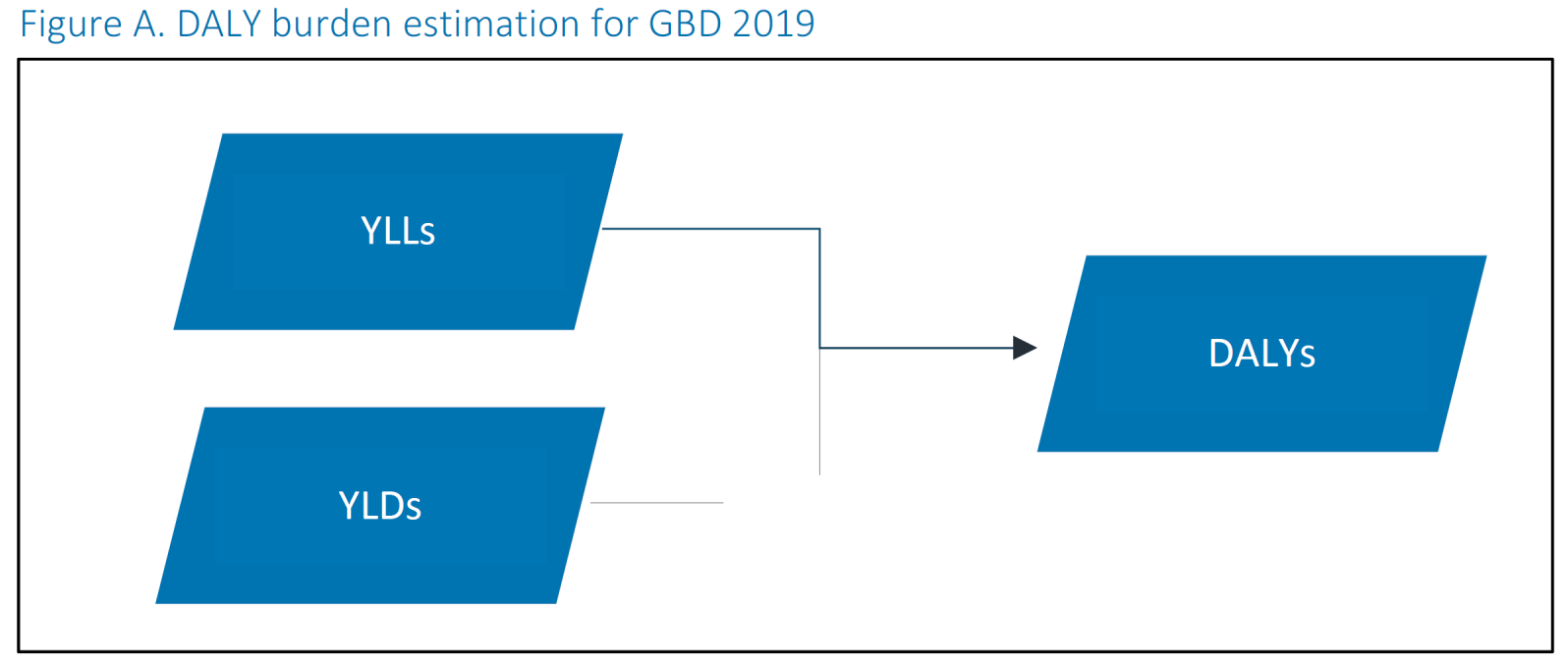


GBD 2019 burden estimation overview

For GBD 2019, we used DisMod MR 2.1 to model the overall prevalence of peripheral arterial disease using prevalence data from literature studies and and crosswalked claims data.

We included the log-transformed, age-standardised SEV scalar for PAD and log-transformed LDI as fixed-effect, country-level covariates. We set value priors of 0 for incidence from ages 0 to 30. We also set a value prior of 0 for remission for all ages. Additionally, we set a value prior of 0 for excess mortality inbetween ages 0 and 30 as well as a value prior between 0 and 0.05 for excess mortality inbetween ages 30 and 100.

**References**

1. GBD 2019 Diseases and Injuries Collaborators. Global burden of 369 diseases and injuries in 204 countries and territories, 1990-2019: a systematic analysis for the Global Burden of Disease Study 2019. Lancet 2020;396:1204-1222.
2. GBD 2017 Disease and Injury Incidence and Prevalence Collaborators. Global, regional, and national incidence, prevalence, and years lived with disability for 354 diseases and injuries for 195 countries and territories, 1990-2017: a systematic analysis for the Global Burden of Disease Study 2017. Lancet 2018;392:1789-1858.
3. GBD 2017 Causes of Death Collaborators. Global, regional, and national age-sex-specific mortality for 282 causes of death in 195 countries and territories, 1980-2017: a systematic analysis for the Global Burden of Disease Study 2017. Lancet 2018;392:1736-1788.
4. GBD 2017 Diet Collaborators. Health effects of dietary risks in 195 countries, 1990-2017: a systematic analysis for the Global Burden of Disease Study 2017. Lancet 2019;393:1958-1972.
5. Peterson HM, Flaxman AD. Meta­regression with DisMod­MR: how robust is the model? Lancet 2013; 381: S110.

**Supplement 2. Methodological details of the age-period-cohort model.**

This study uses an age-period-cohort (APC) model framework to analyse the underlying trends in mortality by age, period, and birth cohort.^1^ The APC model is designed to unpack the contributions of age-associated biological factors, and technological and social factors on disease trends, extending beyond traditional epidemiological analyses.^2^ This approach has been adopted in descriptive epidemiology for certain chronic diseases, including cardiovascular diseases.^3^ Generally, the APC model ﬁts a log-linear Poisson model over a Lexis diagram of observed rates and quantiﬁes the additive effects of age, period, and birth cohorts. As the relationship between age, period, and cohort is perfectly linear (birth cohort = period − age), it is statistically impossible to estimate their independent effects, the so-called identiﬁcation problem.^1,2^ In this study, we circumvent this issue by producing estimable APC parameters and functions without imposing arbitrary constraints on model parameters.^1^

GBD 2019 mortality estimates for PAD and population data of each country/region were used as data inputs for the APC model. In a typical APC model, the age and period intervals must all be equal, ie, ﬁve-year age groups should be used with ﬁve-year calendar periods. As GBD estimates are produced in an unequally spaced data format (ﬁve-year age groups with annual data), we arranged GBD data into a single unit framework by selecting the death and population counts from the mid-year of six ﬁve-year-periods (ie, [1992] 1990 −1994, [1997] 1995−1999 ... [2017] 2015−2019) to represent for the speciﬁc period. The input data included 14 age groups (from 0 to 4 to 65−69 in ﬁve-year age group intervals) and 19 partially overlapping ten-year birth cohorts, as referenced by the mid-year of birth, from 1921 to 1929 (the 1925 cohort) to 2011−2019 (the 2015 cohort). The ﬁtted APC model estimated the overall temporal trend in mortality, which is expressed as the annual percentage change of mortality (ie, the net drift of mortality, % per year). Technically, the net drift is determined by two components: the component of the trend attributable to calendar time and the component of the trend attributable to the successive cohorts. The APC model also estimated the temporal trend of mortality within each age group, expressed as annual percentage change of age-speciﬁc mortality (ie, the local drift of mortality, % per year), and it reﬂects trends in birth cohort effects.^4^ A drift of ±1% per year or more is considered a substantial change in mortality^16^ because this approximates ±10%, ±18%, and ±26% of change in the ﬁtted rate over a period of 10, 20, and 30 years. The signiﬁcance of trends in annual percentage change was tested with a Wald chi-squared test.^4^ The APC model outputs also include ﬁtted longitudinal age-speciﬁc rates in the referent cohort adjusted for period deviations to represent age-associated natural history (ie, age effects), and period (cohort) relative risks of mortality for each period (cohort) to represent period (cohort) effects.^4^ The relative risk is computed as the ratio of age-speciﬁc rates in each period (cohort) relative to reference period (cohort). Both the period (cohort) rate ratio curves incorporate the entire value of the net drift. The choice of referent period (cohort) is arbitrary and does not affect the interpretation of results.

**References**

1. Rosenberg PS, Anderson WF. Age-period-cohort models in cancer surveillance research: ready for prime time? Cancer Epidemiol Bio-mark Prev 2011;20(7):1263–8.

2. Bell A. Age period cohort analysis: a review of what we should and shouldn't do. Ann Hum Biol 2020;47(2):208–17.

3. Zou Z, Cini K, Dong B, et al. Time trends in cardiovascular disease mortality across the BRICS: an age-period-cohort analysis of key nations with emerging economies using the Global Burden of Dis-ease Study 2017. Circulation 2020;141(10):790–9.

4. Rosenberg PS, Check DP, Anderson WF. A web tool for age-period-cohort analysis of cancer incidence and mortality rates. Cancer Epidemiol Biomark Prev 2014;23(11):2296–302.
